# Effects of two isometheptene enantiomers in isolated human blood vessels and rat middle meningeal artery – potential antimigraine efficacy

**DOI:** 10.1186/s10194-019-1003-2

**Published:** 2019-05-03

**Authors:** Alejandro Labastida-Ramírez, Eloísa Rubio-Beltrán, Kristian A. Haanes, René de Vries, Ruben Dammers, A. J. J. C. Bogers, Antoon van den Bogaerdt, Bruce L. Daugherty, Alexander H. J. Danser, Carlos M. Villalón, Antoinette MaassenVanDenBrink

**Affiliations:** 1 Division of Vascular Medicine and Pharmacology, Department of Internal Medicine, Erasmus MC, Dr Molewaterplein 50, 3015 GE Rotterdam, The Netherlands; 2 Department of Neurosurgery, Erasmus MC, Dr Molewaterplein 50, 3015 GE Rotterdam, The Netherlands; 3 Department of Thoracic surgery, Erasmus MC, Dr Molewaterplein 50, 3015 GE Rotterdam, The Netherlands; 4 ETB-BISLIFE, Heart Valve Bank, Zeestraat 29, 1941 AJ Beverwijk, The Netherlands; 5 Tonix Pharmaceuticals, Inc, 509 Madison Avenue, Suite 306, New York, NY 10022 USA; 6 Departamento de Farmacobiología, Cinvestav-Coapa, Czda de los Tenorios 235, Col. Granjas-Coapa, Deleg. Tlalpan, C.P, 14330 Ciudad de México, Mexico

**Keywords:** CGRP, Isolated vessels, Isometheptene, Migraine, Organ baths, Vasodilation

## Abstract

**Background:**

Racemic isometheptene [(*RS*)-isometheptene] is an antimigraine drug that due to its cardiovascular side-effects was separated into its enantiomers, (*R*)- and (*S*)-isometheptene. This study set out to characterize the contribution of each enantiomer to its vasoactive profile. Moreover, rat neurogenic dural vasodilatation was used to explore their antimigraine mechanism of action.

**Methods:**

Human blood vessel segments (middle meningeal artery, proximal and distal coronary arteries, and saphenous vein) were mounted in organ baths and concentration response curves to isometheptene were constructed. Calcitonin gene-related peptide (CGRP)-induced neurogenic dural vasodilation was elicited in the presence of the enantiomers using a rat closed cranial window model.

**Results:**

The isometheptene enantiomers did not induce any significant contraction in human blood vessels, except in the middle meningeal artery, when they were administered at the highest concentration (100 μM). Interestingly in rats, (*S*)-isometheptene induced more pronounced vasopressor responses than (*R*)-isometheptene. However, none of these compounds affected the CGRP-induced vasodilator responses.

**Conclusion:**

The isometheptene enantiomers displayed a relatively safe peripheral vascular profile, as they failed to constrict the human coronary artery. These compounds do not appear to modulate neurogenic dural CGRP release, therefore, their antimigraine site of action remains to be determined.

## Background

Migraine is a neurovascular disorder characterized by recurrent attacks of incapacitating unilateral headaches, recently interconnected with an overall increased risk of stroke and cardiovascular disease [1, 2]. Although its exact pathophysiology has not been elucidated completely, migraine headache has been associated with activation of the trigeminovascular system and increased release of calcitonin gene-related peptide (CGRP), resulting in dysfunctional nociceptive transmission and neurogenic dural vasodilatation [3].

The triptans, serotonergic agonists with selective affinity for 5-HT_1B/1D/(1F)_ receptors, are specific drugs for the acute treatment. Their mechanism of action has been attributed to a dural perivascular inhibition of CGRP release, an inhibition of central nociception and/or a postjunctional constriction of (cranial) blood vessels [4–6]. Because of the latter, the triptans are contraindicated in patients with cardiovascular risk factors or a history of cardiovascular disease.

Isometheptene is a sympathomimetic racemic drug available by prescription or over the counter in several countries, that has long been used for the acute treatment of primary headaches [7, 8]. Nevertheless, a few case reports of acute intracranial vasoconstriction after its use [9, 10] highlight its presumed vasoactive properties [11]. Given that the development of new antimigraine agents with a beneficial cardiovascular safety profile is crucial, Tonix Pharmaceuticals™ separated isometheptene racemate into its enantiomers, (*S*)-isometheptene and (*R*)-isometheptene, a mixed-acting (tyramine-like/minor direct α_1_-adrenoceptor) and an indirect-acting (tyramine-like) adrenergic receptor agonist, respectively. Additionally, (*R*)-isometheptene is an imidazoline I_1_ receptor agonist [12], and previous studies have shown that: (i) imidazoline I_1_ receptor knockout mice have a potentiated nociceptive perception, suggesting that this receptor could be associated with an endogenous analgesia system [13]; (ii) (*R*)-isometheptene decreased trigeminal sensitivity in two rat models of chronic migraine [14]; and (iii) imidazoline I_1_ receptor agonists, like moxonidine and agmatine induced a prejunctional inhibition of the vasodepressor sensory CGRPergic outflow in pithed rats [15]. Together, these findings suggest that a potential antimigraine action of (*R*)-isometheptene could be mediated by inhibition of the trigeminal system. Hence, we hypothesized that the use of only (*R*)-isometheptene will maintain its antimigraine therapeutic effect, while the major side effects associated with the racemate or (*S*)-isometheptene (i.e. cranial vasoconstriction) will be diminished [16].

On this basis, the present study set out to analyse the effects of the isometheptene enantiomers and the racemate on human isolated blood vessels (i.e. middle meningeal artery, proximal and distal coronary arteries, as well as saphenous vein) and trigeminal CGRP-induced neurogenic dural vasodilation in anaesthetized rats (through a closed cranial window).

## Materials and methods

### Human isolated blood vessels

Middle meningeal arteries [internal diameter (ID) 0.5–1.5 mm] were obtained from 11 patients (3 males, 8 females; mean age 53 ± 5 years) who underwent neurosurgical interventions requiring a trepanation of the skull. During surgery, the dura mater together with a small piece of meningeal artery was collected in a sterile organ protecting solution and was immediately transported to the laboratory. The meningeal arteries were dissected and placed in a cold (4 °C) oxygenated Krebs bicarbonate solution with the following composition (mmol/L): NaCl 119, KCl 4.7, CaCl_2_ 1.25, MgSO_4_ 1.2, KH_2_PO_4_ 1.2, NaHCO_2_ 25 and glucose 11.1; pH 7.4.

Saphenous veins (ID 0.5–3 mm) were obtained from 11 patients (10 males, 1 female; mean age 71 ± 2 years) who underwent coronary artery bypass surgery. Immediately after surgery, veins were placed in cold (4 °C) oxygenated Krebs buffer solution with the following composition (mmol/L): NaCl 118, KCl 4.7, CaCl_2_ 2.5, MgSO_4_ 1.2, KH_2_PO_4_ 1.2, NaHCO_2_ 25 and glucose 8.3; pH 7.4.

Proximal (ID 2–3 mm) and distal (ID 0.5–1.0 mm) coronary arteries were obtained from 10 heart valve donors (6 males, 4 females; mean age 40 ± 5 years) who died of non-cardiac disorders: four traumatic brain injury, one benzodiazepine overdose, three anoxic encephalopathy and two cerebrovascular accident. The hearts were provided by the Heart Valve Bank Beverwijk (at that time still located in Rotterdam) from Dutch post-mortem donors, after donor mediation by The Dutch Transplantation Foundation (Leiden, The Netherlands), following removal of the aortic and pulmonary valves for homograft valve transplantation. All donors gave permission for research. Immediately after circulatory arrest, the hearts were stored at 4 °C in a sterile organ protecting solution and were brought to the laboratory within the first 24 h of death. The coronary arteries were dissected and placed in Krebs buffer with the same composition as the one used for the saphenous veins (see above). All blood vessels were used on the same day or stored overnight and used the following day for functional experiments.

The middle meningeal arteries and the distal coronary arteries were cut into ring segments of 1–2 mm length and suspended in Mulvany myographs on two parallel steel wires. The tension was normalized to 90% of the estimated diameter at 100 mmHg [17]. The proximal coronary arteries and saphenous veins were cut into ring segments of about 3–4 mm length and suspended on stainless steel hooks in 15-mL organ baths. The vascular rings were stretched to a stable pretension of 10–15 mN, the optimal pretension as determined earlier [17], and changes in tissue force were measured with an isometric force transducer (Harvard, South Natick, MA, U.S.A.) and recorded on a flatbed recorder (Servogor 124, Goerz, Neudorf, Austria). The buffer was aerated with 95% O_2_ and 5% CO_2_ and was maintained at 37 °C. The segments were allowed to equilibrate for at least 30 min and were washed every 15 min.

### In vitro experimental protocols

Initially, segments were exposed to 30 mM KCl, followed by 100 mM KCl to determine the reference contractile response in each segment. Cumulative concentration response curves were constructed to (*S*)-isometheptene, (*R*)-isometheptene, isometheptene racemate, sumatriptan and noradrenaline, using whole logarithmic steps (1 nM to 100 μM). Sumatriptan and noradrenaline were used as positive controls, as previously reported [17]. Finally, the functional integrity of the endothelium was assessed by observing the relaxation to substance P (10 nM) in arteries or bradykinin (10 μM) in saphenous veins after precontraction with the thromboxane A_2_ analogue U46619 (10–100 nM).

### Animals

Twelve male Sprague-Dawley rats (300–350 g; 8–10 weeks of age), purchased from Harlan Netherlands (Horst, the Netherlands), were maintained at a 12/12-h light-dark cycle in a special room at constant temperature (22 ± 2 °C) and humidity (50%), with food and water ad libitum. Only male rats were used to avoid crosstalk between CGRP and hormonal fluctuations of the oestrus cycle previously described in this model [18, 19]. Experimental protocols were approved by the Erasmus Medical Center’s institutional ethics committee (EMC permission protocol number 3393), in accordance with the European directive 2010/63/EU and the ARRIVE guidelines for reporting experiments in animals [20].

After anaesthesia with sodium pentobarbital (60 mg/kg i.p. followed by 18 mg/kg i.v. per hour), the trachea was cannulated and artificially ventilated (58 strokes/min.; small animal ventilator SAR 830 series, CWE Inc., Ardmore, PA, U.S.A). The adequacy of anaesthesia was judged by the absence of ocular reflexes and a negative tail flick test.

End-tidal pCO_2_ was monitored with a capnograph (Capstar 100 CWE Inc., PA, U.S.A.) and kept between 35 and 45 mmHg. The left femoral vein and artery were cannulated for i.v. administration of drugs and monitoring of mean arterial pressure (MAP), respectively. The animals’ body temperature was maintained at 37 °C by a homeothermic blanket (Harvard Instruments, Edenbridge, Kent, U.K.). The head of each rat was fixed in a stereotaxic frame and the parietal bone overlying a segment of the dural middle meningeal artery was drilled thin, applying cold saline until the artery was clearly visible. As skull drilling induces vasodilation, animals were allowed to rest at least for 1 h before the experimental protocol started. The artery diameter was recorded with an intravital microscopy setup (MZ16, Leica microsystem Ltd., Heerbrugg, Switzerland) using a cyan blue filter on a cold light source. A zoom lens (80-450x magnification) and a camera were used to display images on a standard PC monitor. The artery diameter (30–40 μm at baseline) was continuously monitored and measured with an intravital dimension analyser (IDA 1.2.1.10; U.K.). For periarterial electrical stimulation (ES), a bipolar stimulating electrode (NE 200X, Clark Electromedical, Edenbridge, Kent, U.K.) was placed on the surface of the bone approximately within 200 μm from the artery. The surface of the closed cranial window was stimulated at 5 Hz, 1 ms for 10 s (Stimulator model S88, Grass Instruments, West Warwick, RI, U.S.A.) with increasing voltage until maximum dilation was observed.

### In vivo experimental protocols

After a stable hemodynamic condition for at least 60 min, baseline values of dural artery diameter and MAP were determined. Subsequently, the 12 rats were randomly divided into three sets (*n* = 4 each). In each set, a control vasodilator response of the middle meningeal artery was produced by either endogenous [released by ES (150–300 μA) or capsaicin (10 μg/kg, i.v.)] or exogenous CGRP (1 μg/kg, i.v.). A 30-min interval between control and each subsequent vasodilation was allowed for the recovery of baseline values, and 5 min before the next vasodilation, (*R*)-isometheptene, (*S*)-isometheptene or the racemate (3 mg/kg, i.v., each) were injected. The administration of the isometheptene enantiomers was alternated, and followed by racemate. In each case, there was a time interval of 5 min to allow the dural artery diameter and MAP to return to baseline, before the next vasodilator was administered. We have previously shown that repeated (up to 5 times) ES and treatment with capsaicin or CGRP produced reproducible increases in dural artery diameter (data not shown).

### Statistical evaluation

All data are presented as mean ± SEM. The concentration response curves obtained in the vessels were analysed using GraphPad software (GraphPad software Inc., San Diego, CA, U.S.A.) to calculate the maximal effect (E_max_) and pEC_50_ values. In case a concentration response curve did not reach a plateau, the contraction to the highest concentration was considered as E_max_. E_max_ and pEC_50_ values were compared by unpaired t-test.

The peak increases in dural artery diameter (measured in arbitrary units) in anaesthetised rats are expressed as percent change from baseline. Changes in MAP are expressed in absolute values (mm Hg). A repeated measures one-way analysis of variance (ANOVA) followed by Tukey’s test was performed to examine the different effects per se between isometheptene enantiomers and the racemate. The dural vasodilator differences between the variables within one group were compared using an ANOVA followed by Dunnett’s test. Statistical significance was accepted at *P* < 0.05 (two-tailed).

### Compounds

Apart from the anaesthetic (sodium pentobarbital), the drugs used in the present study were: isometheptene racemate, (*R*)-isometheptene and (*S*)-isometheptene (Tonix Pharmaceuticals Inc., New York, N.Y., U.S.A.); sumatriptan, bradykinin, noradrenaline, capsaicin, U46619 and substance P (Sigma Chemical Co., St. Louis, MO, U.S.A); and rat/human α-CGRP (NeoMPS S.A., Strasbourg, France). Capsaicin was dissolved in a mixture of tween 80, ethanol 70% and water (1:1:8), while the rest of the compounds were dissolved in either distilled water (in vitro*)* or physiological saline (in vivo). The doses mentioned in the text refer to the free base of substances in all cases.

## Results

### Vascular in vitro responses in human middle meningeal artery

**Fig. 1:**
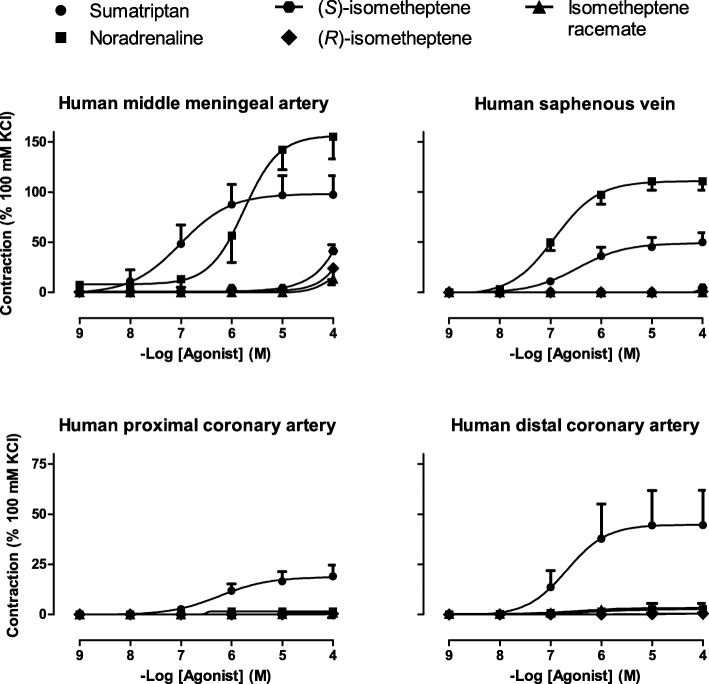
Concentration response curves to sumatriptan, noradrenaline, isometheptene enantiomers and isometheptene racemate on the middle meningeal artery (*n* = 6–7), saphenous vein (*n* = 7–10), as well as proximal (n = 7–10) and distal (*n* = 8–9) coronary arteries

Middle meningeal artery relaxation to substance P (10 nM) was 51 ± 17% of the precontraction induced by U46619. As shown in Fig. 1, sumatriptan induced concentration-dependent contractions that appeared smaller (albeit non-significant) than those induced by noradrenaline at the highest concentrations (E_max_ 98 ± 19 vs. 156 ± 22%; *P* = 0.070; *n* = 6–7). In contrast, the pEC_50_ values were significantly higher for sumatriptan than for noradrenaline (7.0 ± 0.2 vs. 5.8 ± 0.2; *P* = 0.001; n = 6–7).

Interestingly, isometheptene racemate and its enantiomers did not induce any significant contraction, except when they were administered at the highest concentration studied (100 μM, a supratherapeutic concentration), where the enantiomers only induced a modest contraction (20–40% of 100 mM KCl; *P* = 0.002; *n* = 7; Fig. 1).

### Vascular responses in human saphenous vein and coronary artery

In saphenous vein, the endothelium-dependent relaxation to 10 μM bradykinin was 19 ± 4% of the precontraction induced by U46619. Noradrenaline induced concentration-dependent contractions, which were larger and more potent than those induced by sumatriptan (E_max_ 111 ± 9 vs. 51 ± 10%; *P* < 0.001 and pEC_50_ 6.9 ± 0.1 vs. 6.3 ± 0.2; *P* = 0.019; *n* = 9–10). Isometheptene racemate, as well as its enantiomers, did not produce venocontraction, even at the highest concentrations tested (up to 100 μM). In the proximal and distal coronary segments, the endothelium-dependent relaxations to substance P were 31 ± 3 and 83 ± 7% of the precontraction induced by U46619, respectively. As shown in Fig. 1, the contractile responses to sumatriptan and the corresponding pEC_50_ values did not significantly differ between the proximal and distal segments of the coronary artery (E_max_ 19 ± 6 vs. 45 ± 17%; *p* = 0.15 and pEC_50_ 6.3 ± 0.2 vs. 6.2 ± 0.1; *p* = 0.65; n = 9–10), respectively. Noradrenaline was devoid of contractile effects in both artery segments; the same was true for isometheptene racemate and its enantiomers.

### Effect of isometheptene enantiomers and racemate per se on MAP and dural artery diameter in vivo

**Fig. 2:**
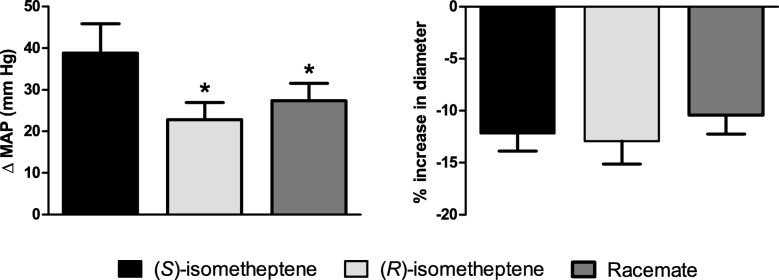
Effect per se of i.v. bolus injections of (*S*)-isometheptene, (*R*)-isometheptene and the racemate (3 mg/kg each) on mean arterial pressure (MAP) and dural artery diameter; all compounds produced significant vasopressor responses and dural vasoconstriction (*P* < 0.05); * *p* < 0.05 as compared to (*S*)-isometheptene

In the closed cranial window experiments, the baseline value of MAP was 92 ± 5 mmHg (*n* = 12). As shown in Fig. 2 (left panel), i.v. injection of both isometheptene enantiomers and the racemate produced significant vasopressor responses (*P* < 0.001; n = 12 each). Remarkably, (*S*)-isometheptene produced more pronounced vasopressor responses than isometheptene racemate and (*R*)-isometheptene (39 ± 7, 27 ± 4 and 23 ± 4 mmHg, respectively; *P* = 0.004; n = 12).

In the dural artery, the administration of (*S*)-isometheptene, (*R*)-isometheptene or isometheptene racemate produced a significant (P < 0.001, n = 12) small, short-lasting decrease in dural artery diameter (12 ± 2%, 13 ± 2% and 10 ± 2% of baseline diameter, respectively; Fig. 2 right panel), which did not differ amongst the agonists (*P* = 0.34; n = 12). The dural artery diameter and MAP values restored to pre-injection levels by the time the next vasodilation was elucidated. At the end of the experiments, the value of MAP (88 ± 5 mmHg) was not significantly different from the initial baseline value (*P* = 0.53; n = 12).

### Effect of ES, capsaicin or CGRP on MAP and dural diameter

In none of the experiments ES (150–300 μA) affected MAP. The i.v. administration of 10 μg/kg capsaicin or 1 μg/kg CGRP produced, as compared to baseline, a similar decrease in MAP of 19 ± 7 and 24 ± 8 mmHg (*P* = 0.68; *n* = 4), respectively. Regarding dural artery diameter, a similar vasodilation was produced after ES and administration of capsaicin or CGRP (62 ± 7, 42 ± 5 and 55 ± 8% of baseline diameter, respectively; *P* = 0.29; n = 4 each).

### Effect of isometheptene enantiomers and the racemate on the dural vasodilatory responses

**Fig. 3:**
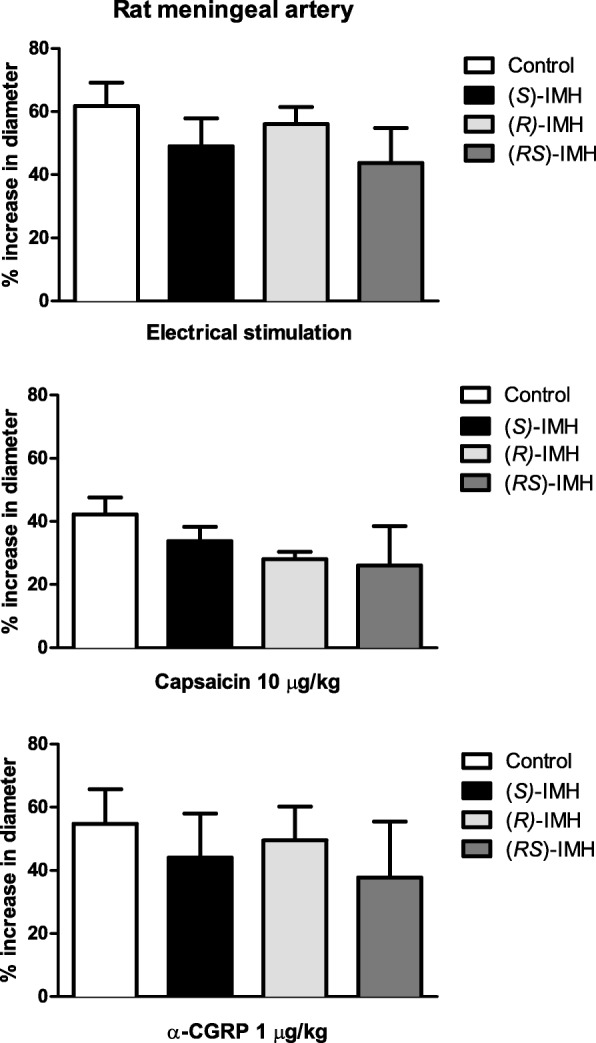
Effect of i.v. bolus injections of isometheptene enantiomers or the racemate (3 mg/kg) on dural vasodilation induced by periarterial electrical stimulation (150–300 μA, upper panels), capsaicin (10 μg/kg, middle panels) or α-CGRP (1 μg/kg, lower panels) in anesthetized rats (*n* = 4 in each group); (*S*)-IMH, (*S*)-isometheptene; (*R*)-IMH, (*R*)-isometheptene; (*RS*)-IMH, isometheptene racemate

After pretreatment with (*S*)-isometheptene, (*R*)-isometheptene or isometheptene racemate, the increases in dural artery diameter (percent change from baseline) evoked by ES (49 ± 9, 56 ± 6 and 44 ± 11%, respectively; *P* = 0.48), capsaicin (34 ± 5, 28 ± 2 and 26 ± 13%, respectively; *P* = 0.42), or CGRP (49 ± 9, 56 ± 6 and 44 ± 11%, respectively; *P* = 0.84) were similar (n = 4 each) to their respective controls in all groups (Fig. 3).

## Discussion

Apart from the implications discussed below, the present study shows the importance of analysing, in an integrative way, the properties of novel antimigraine drugs (namely the isometheptene enantiomers) in different experimental models. Within this context: the use of different human isolated blood vessels allows us to discern possible vascular side effects induced by potential antimigraine agents; and the rat closed-cranial window is an in vivo neurovascular migraine model that focuses on the pathophysiological interaction of the trigeminal system with neurogenic dural vasodilation [21].

### Human vascular (side) effects

A limitation of the current specific antimigraine drugs (i.e. triptans and ergots) is their theoretical risk of coronary vasoconstriction, consequently all vasoactive antimigraine agents are contraindicated in patients with cardiovascular risk factors or coronary artery disease [22]. With this in mind, we investigate the vasomotor effects of the isometheptene enantiomers in different human blood vessels, including the proximal and distal coronary arteries; additionally, the human saphenous vein was included as a positive control of peripheral venoconstriction that is sensitive to α-adrenergic stimulation [23]. Importantly, the isometheptene enantiomers and the racemate were devoid of vasoconstrictor properties in the proximal and distal coronary arteries, as well as the saphenous veins at all concentrations tested.

Similarly, the isometheptene enantiomers were devoid of meningeal contractile effects, except when they were administered at the highest concentration (100 μM, Fig. 1), a supratherapeutic concentration that would never be reached in the clinical situation. Regarding the possible mechanism of action of this meningeal vasoconstriction, it is tempting to speculate that it is mediated by an indirect (tyramine-like) action, resulting in noradrenaline displacement from perivascular sympathetic nerve terminals [24], as previously shown for (*R*)-isometheptene-induced vasopressor responses [25]. Admittedly, we did not test this hypothesis with experiments in the presence of neuronal reuptake inhibitors such as cocaine, because, as mentioned before, this phenomenon only happens at supratherapeutic concentrations and is thus unlikely to be clinically relevant.

In contrast to the meningeal artery, there were no tyramine-like vasoconstrictor effects in coronary arteries, mainly because these vessels (via β_2_-adrenoceptors in vascular smooth muscle) normally dilate to (displaced) noradrenaline [26], as is evident from the lack of contraction after exogenous noradrenaline. Similarly, no tyramine-like responses were observed in saphenous veins; this may be attributed to a lesser sympathetic innervation (vs. arteries) and the possibility that an important amount of perivascular fibres [present in the loose connective tissue surrounding the vein, [27] were destroyed.

Thus, on the basis of these results, the well-established antimigraine action of isometheptene racemate [7, 8, 11], and probably also of isometheptene enantiomers [in particular the antimigraine potential of (*R*)-isometheptene] would seem to be devoid of acute coronary side effects.

### In vivo effects of isometheptene on MAP

In this study, and in accordance with others [25, 28], isometheptene racemate and (*S*)-isometheptene are potent vasopressor compounds in rats. Their vascular responses are mediated by an indirect (tyramine-like action) and a minor direct stimulation of α_1_-adrenoceptors [28]. In contrast, vascular responses to (*R*)-isometheptene are exclusively indirect (tyramine-like action) and of less magnitude than its counterpart enantiomer [25]. Accordingly, (*R*)-isometheptene might produce fewer vascular side effects as an antimigraine agent.

### In vivo effects of isometheptene on dural artery diameter

In contrast to the lack of vasoconstriction in the isolated human middle meningeal artery, the isometheptene enantiomers and the racemate produced equipotent meningeal vasoconstrictor responses in vivo (Fig. 2). This apparent in vitro/in vivo discrepancy suggests that isometheptene’s vasoconstriction is indeed mediated by a tyramine-like action mechanism, which is more evident when the perivascular sympathetic tone is higher (i.e. in vivo), whereas such neurogenic tone has been eliminated in vitro. Although it is believed that the marked dural vasoconstriction of ergots [17] and triptans [29], along with the inhibition of intracranial trigeminal afferents [21] contributes to the peripheral antimigraine mechanisms of these drugs, it is unlikely that isometheptene’s antimigraine action is related to this small, short-lasting decrease in dural artery diameter. Therefore, we proceeded to explore whether the attenuation of experimentally-activated trigeminovascular afferents could explain isometheptene antimigraine efficacy.

### Modulation of perivascular CGRP release as antimigraine treatment

The rat closed cranial window method is a highly predictive model of antimigraine action, in which triptans [21] and CGRP receptor antagonists [gepants and CGRP (receptor)-binding antibodies [30] have shown its ability to inhibit neurogenic (CGRP-mediated) vasodilation of the dural middle meningeal artery as one of their pharmacological sites of action. It is noteworthy that the isometheptene enantiomers and the racemate did not reduce the dural vasodilation evoked by the release of endogenous CGRP (by ES or i.v. capsaicin) or exogenous CGRP (Fig. 3). Hence, inhibition of trigeminal CGRP release, as one of the mechanisms associated with antimigraine action (or vasoconstriction), does not appear to explain isometheptene’s antimigraine efficacy. Interestingly, it has previously been shown that imidazoline I_1_ and α_2_-adrenoceptor agonists are capable of inhibiting prejunctionally the sensory vasodepressor CGRPergic outflow in pithed rats [25, 31]. However, (*R*)-isometheptene, an imidazoline I_1_ receptor agonist with extremely low affinity for α_2_-adrenoceptors [12], did not inhibit the neurogenic dural vasodilation induced by trigeminal stimulation; suggesting a differential receptor expression between sensory and trigeminal afferents, as previously shown for α_2_-adrenoceptors [32]. This suggests that in the rat closed cranial window model, imidazoline I_1_ and α_2_ receptors do not play a role as prejunctional modulators of CGRP release in the trigeminovascular system.

Even though similar sample sizes have been used by two different research groups [32, 33], it could be argued that the statistical power of our in vivo experiments is low (and a limitation) due to the relatively small number of animals used per group. However, when comparing our current results with those of earlier findings using sumatriptan [21] and CGRP antagonists [30], the magnitude of inhibition of CGRP release is remarkably high (up to ca. 70%, and own experiments, data not shown). Thus, while we cannot categorically exclude that a higher number of animals could have produced statistically significant effects, such effects would be rather limited and, probably, devoid of clinical relevance, as an i.v. dose of 3 mg/kg isometheptene is already supramaximal in pithed rats [25].

### Future perspectives for (R)-isometheptene

Isometheptene racemate as monotherapy or, as usual, combined with other drugs (e.g. analgesics), seems a cost-effective alternative (vs. the triptans) in some countries for the acute treatment of mild-to-moderate primary headaches [8, 10]. Whereas (*R*)-isometheptene has been shown not to be effective in the treatment of episodic tension-type headache (https://news-events/news-events/press-releases/detail/1004/tonix-pharmaceuticals-reports-top-line-results-from-phase-2), its antimigraine efficacy has not yet been clinically tested. Overall, after considering the above pharmacological profile of (*R*)-isometheptene, it is not unreasonable to suggest that its potential clinical use as an antimigraine agent may have superior therapeutic advantages over either isometheptene racemate or (*S*)-isometheptene. Most importantly, the fact that (*R*)-isometheptene produced only a slight increase in MAP suggests that it is not directly associated with the intracranial vasoconstriction previously described with the racemate [9, 10].

As an imidazoline I_1_ receptor agonist, (*R*)-isometheptene should possess central antinociceptive properties, as previously shown for other imidazolines agonists [34]. This is supported by a preliminary study where high doses of (*R*)-isometheptene decreased trigeminal sensitivity in two rat models of chronic migraine, and this effect was associated with a reduced CGRP immunoreactivity in the trigeminal nucleus caudalis. Certainly, further experiments, falling beyond the scope of the present study, will be required to investigate whether: (i) (*R*)-isometheptene is capable of inducing central antinociception; (ii) activation of imidazoline receptors translates into acute or prophylactic antimigraine action; and (iii) selective imidazoline I_1_ receptor agonists can be developed as novel antimigraine agents.

## Conclusion

It is noteworthy that the isometheptene racemate and its enantiomers displayed a relatively safe peripheral vascular profile, as they failed to constrict the human coronary artery. Isometheptene’s antimigraine action appears unrelated to modulation of the trigeminovascular system and CGRP release, but most likely involves central mechanisms. The exact site and mechanism for antinociceptive modulation still remains to be elucidated.

## Abbreviations

CGRP: Calcitonin gene-related peptide
ES: Electrical stimulation
i.p: Intraperitoneal
i.v: Intravenous
ID: Internal diameter
MAP: Mean arterial pressure
NA: Noradrenaline
